# Proteolytic Remodeling of Perineuronal Nets: Effects on Synaptic Plasticity and Neuronal Population Dynamics

**DOI:** 10.1155/2018/5735789

**Published:** 2018-02-04

**Authors:** P. Lorenzo Bozzelli, Seham Alaiyed, Eunyoung Kim, Sonia Villapol, Katherine Conant

**Affiliations:** ^1^Department of Neuroscience, Georgetown University Medical Center, 3800 Reservoir Rd NW, Washington, DC 20007, USA; ^2^Interdisciplinary Program in Neuroscience, Georgetown University Medical Center, 3800 Reservoir Rd NW, Washington, DC 20007, USA; ^3^Department of Pharmacology, Georgetown University Medical Center, 3800 Reservoir Rd NW, Washington, DC 20007, USA

## Abstract

The perineuronal net (PNN) represents a lattice-like structure that is prominently expressed along the soma and proximal dendrites of parvalbumin- (PV-) positive interneurons in varied brain regions including the cortex and hippocampus. It is thus apposed to sites at which PV neurons receive synaptic input. Emerging evidence suggests that changes in PNN integrity may affect glutamatergic input to PV interneurons, a population that is critical for the expression of synchronous neuronal population discharges that occur with gamma oscillations and sharp-wave ripples. The present review is focused on the composition of PNNs, posttranslation modulation of PNN components by sulfation and proteolysis, PNN alterations in disease, and potential effects of PNN remodeling on neuronal plasticity at the single-cell and population level.

## 1. Extracellular Matrix in the Brain

Within the central nervous system (CNS), there are three main types of extracellular matrix (ECM). A homogenous, hyaluronic acid- (HA-) based, relatively loose form of ECM enwraps cell bodies, dendrites, and synapses of most neurons within the brain and may serve as a reservoir for proteins including thrombospondins and guidance molecules [1, 2]. The extracellular portion of membrane-tethered adhesion molecules represents the second form of ECM. This subtype may be substantially remodeled as a function of neuronal activity or injury, and the contribution of such to activity-dependent plasticity is the subject of several recent reviews [3–8]. The present review concentrates on the third type of ECM that is subject to activity-dependent modulation, a relatively rigid and unique lattice-like structure that envelops the soma and proximal dendrites of select neuronal subpopulations and is commonly referred to as the perineuronal net (PNN) [8–13]. As compared to loose ECM, the PNN is aggrecan- and chondroitin sulfate-enriched [1].

**Figure 1:**
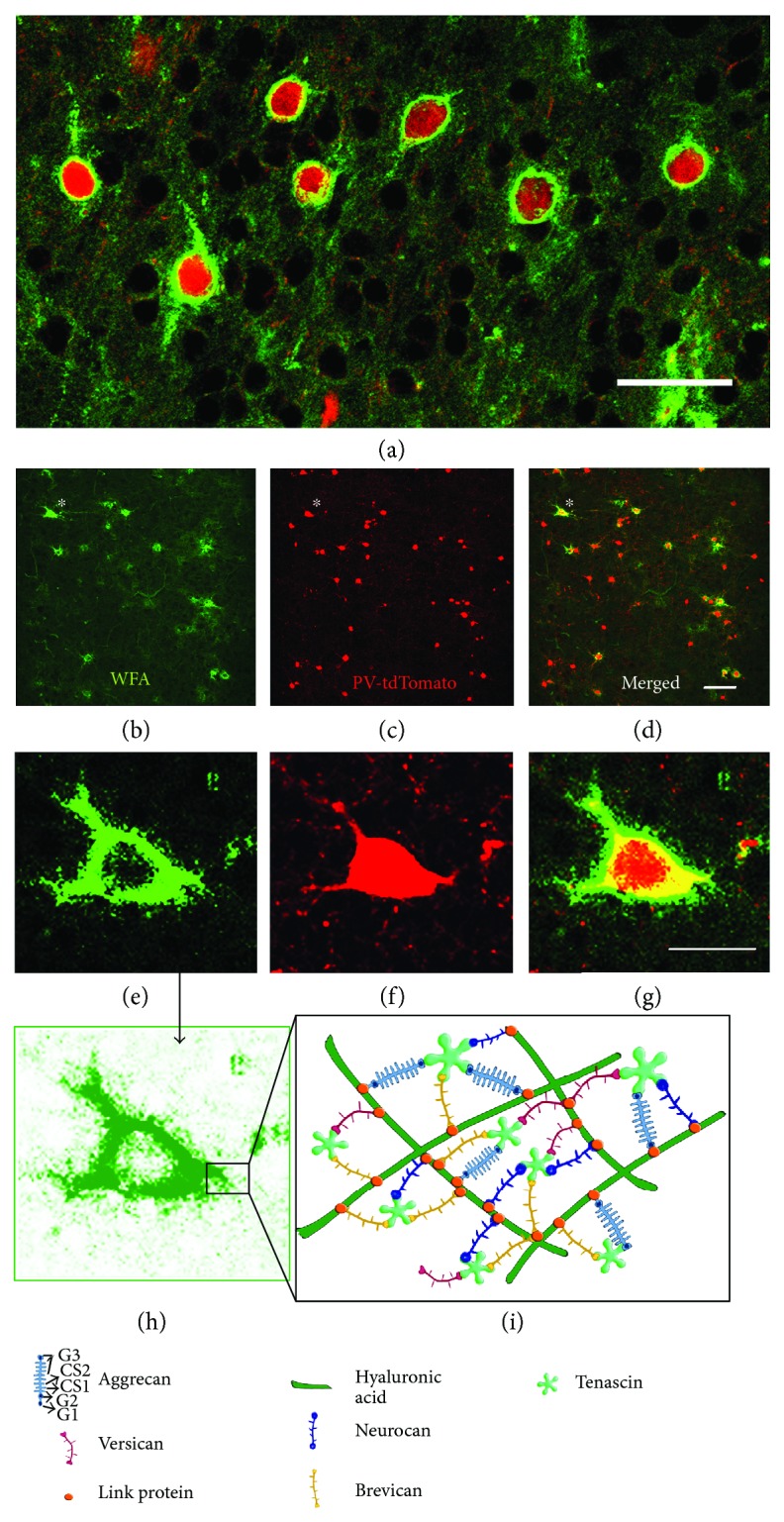
Colocalization of perineuronal nets with parvalbumin-expressing neurons shown in (a)–(c) is a region of interest from the prefrontal cortex of a PVCretdTomato mouse. Mice were generated through crosses between PV-Cre (B6.129P2-Pvalbtm1(cre)Arbr/J; JAX #008069) driver and tdTomato (Ai14; JAX #07914) reporter animals and bred so as to avoid the confound of ectopic expression [19]. In (e)–(g), closeup views are shown from the cells noted by asterisks in (b)–(d). This 30 *μ*m slice was incubated with fluorescein-labeled (green) *Wisteria floribunda* lectin (WFA) (1 : 1000, Vector Laboratories, FL-1351). WFA labels PNNs, which can be observed along the soma and proximal dendrites of several PV neurons shown in low-power views and appreciated along the same regions in the high-power image of one neuron (g) (scale bar: (a)–(c), 50 *μ*m; (e)–(g), 20 *μ*m). Shown in (d) is representative PNN staining in the murine sensory cortex. The schematic, in which individual PNN components are highlighted, is shown in (h).

In terms of the overall structure, the PNN is composed of a linear nonsulfated hyaluronic acid polymer that forms the backbone of the lattice. Chondroitin sulfate proteoglycans (CSPGs), including the lecticans aggrecan, versican, neurocan, and brevican, are joined to the hyaluronic acid polymer through “link” proteins including cartilage link protein (Crtl1). Tenascins bind to the C-terminal domains of CSPGs to complete the complex structure of the lattice [14], with tenascin-R likely an indispensable component [15, 16]. In Figure 1, we show the PNN staining in the murine cortex and the schematic highlighting varied components of the lattice.

PNNs are expressed in varied brain regions including the cortex, hippocampus, amygdala, hypothalamus, basal ganglia, and cerebellum. In the prefrontal cortex, the PNN is highly colocalized with PV GABAergic interneurons [17]. Expression is particularly robust in deeper cortical layers [17]. While colocalization with cortical pyramidal cells is less common, this does occur and is more often observed in the visual cortex than in secondary motor areas [18]. PNNs have also been identified to surround excitatory neurons of the deep cerebellum [20–23]. In CA1 and CA3 regions of the hippocampus, PV cells again represent the majority of ensheathed cells. In the CA2 region, however, PNNs are frequently observed to surround excitatory pyramidal cells [17, 24].

Species differences have also been described with respect to PNN deposition [17]. For example, while low levels of PNN staining have been noted in the rat basal ganglia, high levels have been noted in that of mice [20, 21, 25]. Intriguingly, in terms of the PNN to contribute to behavioral differences across species, recent work suggests that PNN deposition is increased in songbird species with closed-end song learning as opposed to those that show extensive adult vocal plasticity [26].

## 2. Regulation of PNN

### 2.1. PNN and Component Expression as a Function of Development and Cell Type

Deposition of PNN typically increases with neuronal activity and brain maturation [27]. A shift in ECM composition also occurs during development, with neurocan and tenascin-C expression being notable at late embryonic stages but downregulated during adulthood [1]. Tenascin-R and CSPGs such as aggrecan generally represent principal PNN components in the mature brain [1].

Both neurons and glial cells express specific components of the structure. *In vitro* work has shown that neurons express the CSPG aggrecan and that expression is substantially upregulated with depolarization [28]. Aggrecan expression is instead downregulated during various forms of sensory deprivation during development, due to a reduction in aggrecan transcripts, also suggesting that its expression is activity-dependent [29–31]. Expression of PNN components by PV neurons in particular is intriguing in a way that it may be regulated in significant part by the homeobox protein orthodenticle homeobox-2 (Otx2) [32]. This protein is thought to be released from cells including those of the choroid plexus [33] and taken up by PV cells to influence their maturation and PNN expression. In a proposed positive feedback loop, Otx2 can then bind to the expressed PNN in a manner that facilitates its uptake [34, 35].

Neurons can also express HA when cultured in the absence of glia. Nonetheless, glia are an important source of HA and are important for the expression of PNN components including brevican and hyaluronan, as well as proteoglycan link protein 1 (HAPLN1) [28]. In nonneural cells, soluble factors including platelet-derived growth factor have been shown to increase expression of hyaluronan synthases [36]. Alternatively, inhibition of specific protein kinases (MEK1/2 and PI3K) results in a decrease in hyaluronan synthase 2 transcript and activity levels [36].

### 2.2. Changes in PNN Integrity as Related to Neural Plasticity and Critical Periods

Intriguingly, maturation of the PNN has been associated with the closure of critical periods of CNS plasticity (reviewed in [37, 38]). These critical periods have been described for varied forms of learning. Expanded cortical representation of a stimulated whisker can occur prior to critical period closure, as can language acquisition [37]. Similarly, altered central responsiveness to an occluded eye or deprived (cauterized) whisker can occur prior to closure, as can rapid remodeling of axonal arbors in the visual cortex [30, 39, 40]. The timing at which complex PNN deposition occurs coincides with the end of the critical period in the visual cortex, and PNN digestion with chondroitinase ABC can reopen plasticity [39]. Moreover, dendritic spines of the adult visual cortex show increased structural and functional plasticity following chondroitinase treatment [41]. Consistent with the role for neuronal activity-dependent PNN deposition in the ultimate closure of critical periods of cortical plasticity, sensory deprivation may delay the same [37].

PNN integrity also controls plasticity in the amygdala and hippocampus. In the amygdala, PNN deposition is followed by fear memory persistence and chondroitinase treatment can render fear memories susceptible to erasure [42]. In the hippocampus, chondroitinase treatment renders the normally LTP-resistant CA2 to the CA3 pathway susceptible to the same [24]. This study also noted that in hippocampal CA2, PNN-enwrapped pyramidal neurons were the targets of chondroitinase treatment. In terms of the PNN and additional subcortical regions, improved performance in water maze learning has been associated with maturation of PNN within the striatum [43].

**Figure 2:**
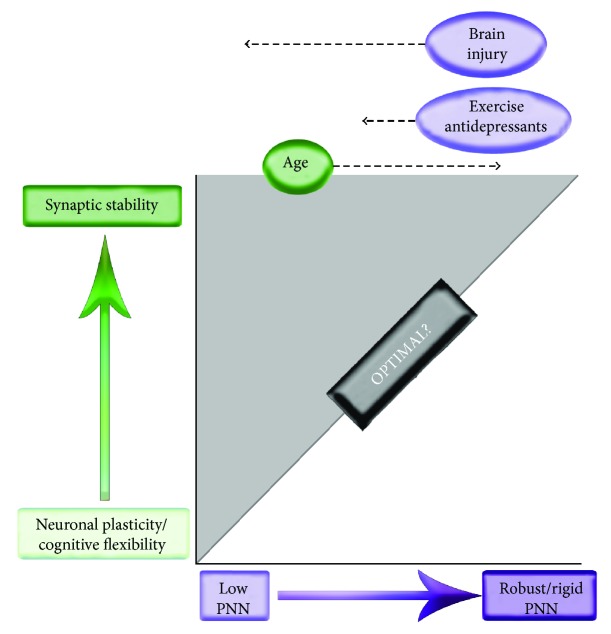
Hypothetical relationship between PNN expression and synaptic stability. PNN deposition increases with development and may further increase with aging. Developmental deposition is linked to closure of critical periods of plasticity and excessive postdevelopment; late-age-related deposition may ultimately limit cognitive flexibility. Acute brain injury can lead to a substantial loss of PNN with consequent effects on the function and viability of PV-expressing neurons. Antidepressant medications and exercise, which may more modestly increase the levels of PNN-degrading proteases [44, 45], could potentially reduce PNN density to a lesser extent, so as to increase cognitive flexibility while sparing interneurons from injury.

Overall, PNN deposition appears to be important to structural stability of the neuronal circuitry that underlies long-term memory. Reductions in PNN integrity may instead be associated with improved cognitive flexibility [1]. Together, these observations suggest that there may be an optimal level of PNN density (see Figure 2). Interestingly, select mouse models of Alzheimer's disease (AD) show increased PNN deposition which is associated with impairments in hippocampal long-term potentiation [46].

### 2.3. Physiologically Relevant Posttranslational Modification of the PNN

In terms of flexibility, PNN abundance is modulated by proteolytic processing. While experimental studies often utilize hyaluronidase or chondroitinase to affect PNN degradation [24, 47, 48], MMPs and ADAMTS proteins likely represent important physiological modulators of the PNN [1]. This is supported by recent work showing that PNN is more abundantly expressed in juvenile MMP-9-null mice [49]. Similarly, the endogenously expressed serine protease tissue-type plasminogen activator has the potential to reduce PNN abundance as supported by work showing that its injection into the visual cortex can prolong or reactivate ocular dominance plasticity [50].

Numerous studies have investigated the role for specific proteases as effectors of PNN component processing. Both ADAMTSs and MMPs are able to degrade aggrecan [51–53]; however, ADAMTSs may be more efficient than MMPs with respect to the cleavage of the aggrecan core protein [54, 55]. Brevican is also degraded by various MMPs and ADAMTS proteases [56, 57], including ADAMTS-4 [58] and ADAMTS-5 [59]. There may, however, be a regional specificity regarding ADAMTS-dependent cleavage of brevican given the lack of colocalization between WFA-labeled PNNs and ADAMTS-derived brevican cleavage fragments in the rodent brain [60]. Additionally, the role of other enzymes in aggrecan and brevican proteolysis cannot be ruled out since cleavage products of both proteins are detected in Adamts-4^−/−^ and Adamts-5^−/−^ mice following spinal cord injury [61]. In contrast, versican cleavage is not observed in either knockout mouse following injury [61].

Though ADAMTS proteases and MMPs may differ in terms of overall substrate specificity, in the case of shared lectican substrates, they can cleave at distinct sites. This has allowed for the investigation of enzyme-specific cleavage based on the different neoepitopes that are formed (reviewed in [62]).

Lectican-degrading enzymes have been shown to be expressed and/or activated in a temporally dependent manner which often parallels neuronal development [63]. For instance, the lectican-degrading ADAMTS-15, which is expressed exclusively by PV+ interneurons, displays the highest expression during synaptogenesis—a period which precedes the formation of PNNs [63]. In addition, the MMP-2 substrate neurocan, which was initially named 1D1, undergoes increased proteolysis during rat development [64].

CSPG sulfation can also influence plasticity [65–67] and protease-dependent PNN processing [68]. Sulfation by 4-O-sulfotransferase-1 (C4ST-1) or 6-O-sulfotransferase-1 (C6ST-1) to produce chondroitin sulfate with 4-O-sulfation (C4S) or 6-O sulfation (C6S) has been described [68]. The C6S form, which is more susceptible to proteolysis by select ADAMTS proteases [68], decreases in relative abundance with brain aging [69]. Interestingly, transgenic mice that overexpress C6ST-1 show a juvenile level of ocular dominance plasticity into adulthood [66].

Proteolytic processing of PNN has broad implications in terms of neurological disease and therapeutics. Indeed, PNN degradation has been observed in association with seizure activity, CNS infection, cerebral ischemia, and traumatic brain injury [70–73]. A less dramatic reduction, which might instead be beneficial to brain function, has been observed in the background of treatment with select antidepressant medications [74]. In the following sections, we focus on select pathological conditions in which alterations in PNN integrity have been described.

## 3. PNN Changes in Pathology

### 3.1. Human Immunodeficiency Virus (HIV)

Though HIV infects CD4-expressing lymphocytes and monocyte-derived cells, associated CNS dysfunction is thought to be driven in large part by the latter [75]. Neurons themselves are not infected by the virus but are subject to injury from the products of infected microglia and macrophages [75]. These products include proinflammatory cytokines and high levels of PNN-degrading proteases [76]. We and others have shown that varied MMPs are substantially upregulated in the HIV [76–80] and simian immunodeficiency virus (SIV) brain [81]. In addition, two separate disintegrins and metalloproteinases with thrombospondin motifs (ADAMTS-1 and ADAMTS-4) are highly expressed in an SIV model [82]. Consistent with this, PNNs are substantially reduced in the brains of HIV-infected individuals and virtually absent in cases of HIV encephalitis as compared to controls [72]. In an SIV model, PNN integrity is also reduced [82]. Recently, inhibitory interneurons, including parvalbumin-positive (PV+) neurons, were found to be particularly susceptible to injury mediated by the HIV-1-encoded Tat protein [83]. Tat promotes MMP expression [84, 85], and it is thus tempting to speculate that MMP-stimulated degradation of PNNs contributes to the injury of PV interneurons.

### 3.2. Alzheimer's Disease (AD)

Analyses of PNN integrity in AD have yielded contradictory findings, which may be due to complex interactions between degrading enzymes and their inhibitors, differences in ECM detection methods, or differences in mouse models that have been studied. ECM proteins such as the heparan sulfate proteoglycan (HSPG) agrin have been associated with senile plaques (SPs) and neurofibrillary tangles (NFTs) [86, 87]. Specific chondroitin sulfate proteoglycans (CSPGs) are also found in SPs and NFTs [88]. Some ECM proteins, such as brevican, are elevated in the AD brain [89] while other ECM components detected by *Wisteria floribunda* (WFA), such as N-acetylgalactosamine, are decreased [90]. To further complicate the issue, one study concluded that perineuronal nets were largely unchanged in the AD brain [91]. Interestingly, PNN+ neurons were almost never associated with NFTs [91]. This finding is in agreement with the suggestion that PNNs protect neurons against AD-related aberrant protein accumulation [92–95].

Literature regarding PNNs in AD mouse models has also provided conflicting results. PNNs have been found to be largely unaffected in the Tg2576 mouse model of AD when using both aggrecan immunoreactivity and WFA staining; however, in the APP/PS1 tg mouse, total protein levels of neurocan, brevican, and tenascin-R are increased as is the number of WFA-labeled PNNs [46]. Interestingly, two separate studies using APP/PS1 mice at either 3 months or 15 months of age observed reduced amyloid pathology and increased synaptic protection, following intrahippocampal injections of chondroitinase ABC (ChABC) [46, 89]. ChABC injections also rescued deficits in contextual memory and restored long-term potentiation [46].

### 3.3. Seizure Activity

Extensive PNN alterations have been observed in epilepsy models [96]. Aggrecan expression is transiently increased in the brain two days after a kainic acid- (KA-)induced seizure [97], and the expression later returns to control levels. In addition, the juvenile form of the CSPG neurocan is also transiently elevated in the brain after seizure [98, 99]. In a domoic acid model of epilepsy, upregulation of neurocan and tenascin-C was observed in the first week, followed by an increase in phosphacan a week later when recurrent seizures occurred [100]. Brains of temporal lobe epilepsy patients who exhibit Ammon's horn sclerosis show that tenascin-C is increased in the hippocampus and that tenascin-C undergoes a redistribution in which expression patterns become disrupted [101]. In a pilocarpine model of epilepsy, an initial decrease in heparan sulphate expression is observed in the acute phase, which is then followed by increased expression of chondroitin sulphate during subsequent seizure-free and chronic seizure periods [102]. Further complicating this, however, are findings that phosphacan levels decrease after kainate application [98, 99] and phosphacan levels are also reduced in the dentate gyrus of 8-month-old Ihara epileptic rats [103].

Reported reductions in ECM are largely thought to be due to increased MMP activity given that MMP-9 levels increase within hours of kainate application [104, 105]. Increased serum levels of MMP-9 and decreased levels of tissue inhibitor of metalloproteinase 1 (TIMP-1) were found in children who experienced acute encephalopathy following prolonged febrile seizures [106]. Studies that have manipulated mouse genetics further support the role of MMP-9. For example, sensitivity to pentylenetetrazole (PTZ) epileptogenesis is reduced in the MMP-deficient background, while MMP-9 overexpression instead can increase sensitivity to the same [107]. Moreover, MMP-dependent degradation of aggrecan is observed in a PTZ model, and recent work has identified additional MMPs, MMP-3 and MMP-13, as being increased in a pilocarpine model [108]. MMP inhibition prevents perineuronal net breakdown and also reduces seizure induction in a kindling mouse model [109]. Others have observed increased ADAMTS-dependent cleavage of the CSPG brevican after kainate application [110]. Modulation of protease activity following seizure could represent an avenue to pursue towards neuroprotection.

### 3.4. Stroke and Traumatic Brain Injury

Reductions in PNN staining have been observed with focal cortical photothrombotic stroke injury in rats [111]. Moreover, in a model of permanent middle cerebral artery occlusion (MCAO), a loss of WFA-binding matrix components in the cortical infarct core is observed, and this is associated with neuronal death [73]. Downregulation of PNNs in ischemic brains may in turn create conditions favorable for synaptic remodeling [112]. Of interest, enriched housing after stroke leads to a significant loss of PNN immunoreactivity, specifically with a robust reduction in aggrecan-containing PNNs surrounding PV-expressing GABAergic neurons in the ipsilateral cortex [113].

A loss of PNN and parvalbumin immunoreactivity is also observed in models of traumatic brain injury (TBI). In rodent models, PNN immunoreactivity is reduced in the perilesional cortex following fluid-percussion injury [71] or controlled cortical impact injury [114]. Accumulation of Otx2 in PV-expressing cells is critical for PNN maintenance [34], and Otx2 levels and PNN integrity are reduced following TBI. This suggests that Otx2 could represent a promising treatment for brain injury [71]. Recent work has demonstrated that TGF-*β* may regulate extracellular matrix remodeling and the PNN in a rat model of TBI, as well in brains of human epileptic patients [115]. Albumin also leads to PNN degradation, with TGF-*β* signaling again playing an important role [115].

### 3.5. Depression

Alterations in PNN staining have been observed in patients with depression [12, 116]. For example, a decrease in C6S PNNs, which are more sensitive to proteolysis, has been observed in the amygdala of those with bipolar depression [117]. Polymorphisms in the PNN expression regulator Otx2 also have been linked to bipolar disorder [118]. Moreover, a neurocan-knockout mouse shows behavioral changes that are consistent with mania, including increased saccharin preference, hyperactivity, and reduced immobility time in the forced swimming test [119].

Additionally, monoamine antidepressants have been linked to alterations in PNN. For example, chronic treatment with fluoxetine decreases PNN staining in the murine medial PFC and hippocampus [120]. Fluoxetine exposure in utero can also reduce postnatal PNN deposition in the murine amygdala and hippocampus [121]. Importantly, monoaminergic signaling may increase expression and activity of PNN-degrading proteases [122]. Consistent with this possibility is work demonstrating increased MMP-9 expression in venlafaxine-treated rats [45] and increased MMP expression in ex vivo cells treated with norepinephrine (S. Alaiyed et al., unpublished observations, and [123]).

Lithium, an effective treatment for bipolar illness, has also been associated with alterations in the PNN and more specifically with CSPG digestion [124, 125]. In addition, lifetime exposure of bipolar patients to lithium is associated with an increase in PNN sulfation patterns that render the nets more sensitive to proteolytic digestion [117].

In terms of the significance of PNN remodeling in the setting of antidepressant treatment, since reduced PNN integrity may reduce PV interneuron excitability to increase overall excitatory/inhibitory balance [47, 126], which is reduced in major depression [127], PNN remodeling might contribute to antidepressant efficacy.

## 4. Function of the PNN

PNNs likely serve varied nonmutually exclusive functions. Constituent components impart nets with an overall negative charge [128], which may in turn affect the local electrical field sensed by gating subunits of ion channels [129]. In combination with other effects of the PNN, changes in ion channel gating may influence PV excitability [129]. The anionic nature of the net may also facilitate sequestration of cations including Na^+^ and K^+^ [130, 131], as well as positively charged potentially toxic metal ions [132, 133]. The latter may include iron, which can undergo intracellular import and stimulate oxidant stress. In support of the potential for an intact PNN to protect cells from oxidant stress is research showing that PNN degradation increases susceptibility of PV cells to injury [71].

An intact PNN can also serve to anchor bioactive molecules including growth factors and cytokines. This could be adaptive or nonadaptive depending on the extent of remodeling and physiological or pathological background on which it is occurring. With respect to the former, PNN digestion can facilitate enhanced axonal sprouting [134–136]. If enhanced axonal sprouting has a differential effect with respect to inhibitory or excitatory inputs to PV interneurons, cortical excitation may be altered. As a related concept, basket cells with a low excitatory to inhibitory synaptic density ratio are observed with enrichment [137], and disinhibition that follows reduced PV activity from varied causes has been described in learning paradigms and linked to increased cortical excitability [138].

Several studies suggest that the PNN contributes to the stability of preformed synapses. Elegant work has shown that PNN disruption allows for increased lateral diffusion of glutamate receptor subunits [139]. The diffusion constant of GluA1 drops as the subunit reaches a hyaluronan-enriched area. Hyaluronidase digestion increases both the diffusion coefficient and the total surface area covered by individual subunits [139]. In addition, synaptically released glutamate may show increased diffusion in the absence of a perineuronal net. Since the PNN likely surrounds glutamatergic inputs along the soma and proximal dendrites of PV neurons, PNN disruption has implications for excitatory input to this population and thus their ability to inhibit pyramidal cell firing. Recent work from the Favuzzi group has demonstrated that the somas of PV cells that are surrounded by brevican-positive nets receive more excitatory input from pyramidal cells than those without brevican-positive nets do [126]. Moreover, loss of brevican reduces EPSCs but not IPSCs recorded from PV cells [126]. Chondroitinase-mediated net digestion has also been linked to reductions in IPSCs recorded in pyramidal cells, suggesting that loss of PNN may reduce PV output [140].

Overall, it appears that an important function of the PNN is to restrict plasticity and instead support synaptic stability (this concept is reviewed in [1]). Consistent with this, PNN disruption or reduced PNN maturation has been linked to improved cognitive flexibility. For example, tenascin-R-knockout animals show normal spatial acquisition during water maze learning, but acquired memory is more vulnerable to erasure during a subsequent reversal phase [141]. Animals that lack the link protein Crtl1 have attenuated nets and persistent plasticity in the visual system [142]. In addition, increased PNN integrity within the amygdala has been linked to erasure-resistant fear memory [42]. Moreover, ECM degradation mediated by bilateral auditory cortex injection of hyaluronidase accelerated relearning in a tone discrimination task [143]. A hypothetical overview of the relationship between PNN deposition and synaptic stability is shown in Figure 2.

Though we have thus far concentrated our discussion of PNN-enwrapped PV cells, which represent the predominantly ensheathed neuronal population in the sensory cortex as well as in the CA1 and CA3 hippocampus, it should be noted that in regions including hippocampal CA2, the PNN also envelops pyramidal cells [17, 24]. Interestingly, chondroitinase treatment of ex vivo hippocampal slices makes the normally LTP-resistant CA3 to the CA2 pathway display LTP [24]. Chondroitinase did not, however, affect basal excitatory transmission in CA2 pyramidal cells [24].

## 5. Implications of Altered PNN Integrity at the Network Level

Given that PV-expressing interneurons represent the predominant neuronal subtype that is enveloped by PNNs, changes in PNN integrity likely have a substantial effect on PV-stimulated neuronal activity. Along with cholecystokinin- (CCK-) expressing cells, the PV subpopulation represents one of the two major GABAergic basket cell subtypes. As compared to CCK-positive cells, PV-positive cells receive strong excitatory input, are fast firing, and can release GABA in a relatively reliable fashion. For these reasons, PV neurons represent pacemakers for synchronous network events [144–146].

Emerging evidence supports the role for PNN alterations in synchronous network activities such as cortical gamma oscillatory activity. For example, a recent study demonstrated that PNN removal could decrease activity in inhibitory units as inferred from their waveforms [47]. In association with this disinhibition, PNN removal also increased gamma activity in the visual cortex [47]. If the PNN is indeed important to glutamatergic input to PV neurons, then, this study is in line with others that have more selectively reduced the same. In the work by the Racz group, mice were engineered so that AMPA-mediated excitation of PV interneurons was selectively suppressed. The high-frequency discharge of pyramidal cells was facilitated in these animals [147].

Though PNN staining in the hippocampus is less robust than that in the visual cortex [17], reduced PV activity could nonetheless influence population activity in this brain region as well. In recent work, we have observed that ex vivo treatment of hippocampal slices with hyaluronidase or chondroitinase can increase the frequency of sharp-wave ripple (SWR) events [48]. The sharp wave reflects depolarization of CA1 pyramidal cells by synchronous discharge of CA3 axonal inputs [148]. CA1 PV cells contribute to high-frequency ripple oscillations [148]. Hypothetically, a reduction in PV cells that are enwrapped by an intact PNN could limit their glutamatergic input [139] and thus their ability to inhibit activity of the principal cells that initiate events.

The potential for modulation of the PNN to affect gamma rhythms has physiological implications. Gamma activity is thought to provide a temporal structure for information processing [149]. It may also contribute to and/or reflect altered excitatory inhibitory balance as related to mood. Consistent with this possibility, GABAergic interneuron-mediated cortical inhibition has been linked to reduced gamma activity [150], and gamma is also diminished in models of depression [151]. Remission of depressive symptoms induced by chronic restraint stress correlates with restoration of gamma activity [151].

Similarly, effects of PNN modulation on SWR frequency are of potential interest. SWRs represent a population event in which sequential replay of previously activated neuronal assemblies occurs in a time-compressed manner [152, 153]. This replay of assemblies, initially activated during spatial learning [154], is thought to be critical for information transfer and memory consolidation. SWR density is increased after learning, and disruption of SWRs during sleep impairs subsequent memory of presleep learning [155, 156].

It should be noted that while limited remodeling of the PNN might have beneficial effects on CNS function, excess remodeling might instead enhance seizure susceptibility. Consistent with this, published work suggests that PNN disruption can induce hyperexcitability and epileptiform-like activity in cultured hippocampal networks [157, 158]. Moreover, a loss of the PNN following a traumatic lesion was associated with reduced inhibitory tone, leading the authors to suggest that PNN disruption might contribute to posttraumatic epilepsy [71].

## 6. Summary

Despite their cage-like appearance and lengthy backbones, perineuronal nets are relatively plastic structures that can be modulated by sulfation and proteolysis. At the single-cell level, PNNs may limit diffusion of released transmitters and transmembrane receptors to influence the efficacy of synaptic transmission. Importantly, however, factors that are known to influence PNN integrity including infectious agents, ischemia, and select therapeutics tend to affect PNN integrity in a widespread manner which may in turn have important effects on neuronal population dynamics [47, 71]. Given that population dynamics are critical for mood, attention, and memory, additional studies of the PNN modulation and synchronous population activity are warranted.
